# Safety Profile of Amnion-Derived Cellular Cytokine Solution (ACCS) Following Topical Skin Application in Patients Receiving Breast Radiotherapy

**Published:** 2015-07-28

**Authors:** Mark Trombetta, Thomas B. Julian, D. Lawrence Wickerham, David L. Steed

**Affiliations:** ^a^Department of Oncology, Allegheny General Hospital, Pittsburgh, Pa; ^b^Department of Surgery, Allegheny General Hospital, Pittsburgh, Pa; ^c^Drexel University College of Medicine, Allegheny Campus, Pittsburgh, Pa; ^d^National Surgical Adjuvant Breast and Bowel Project, Pittsburgh, Pa; ^e^Stemnion, Inc, Pittsburgh, Pa

**Keywords:** radiation, skin reaction, breast, cytokine, dermatitis

## Abstract

**Objective:** To establish a safety profile for amnion-derived cellular cytokine solution following topical application in patients undergoing whole breast radiotherapy for breast cancer. **Materials and Methods:** Twenty female patients with early-stage breast cancer were enrolled in 2 separate cohorts of an institutional review board–approved phase I protocol. Cohort 1 consisted of 10 patients who received topical amnion-derived cellular cytokine solution to the breast immediately following the first 10 fractions of whole breast radiotherapy. Cohort 2 consisted of 10 additional patients who fit the same criteria as the initial cohort but received topical amnion-derived cellular cytokine solution following the development of at least grade I breast erythema. Blood samples were tested for the presence of proteins in amnion-derived cellular cytokine solution as well as routine hematologic functions. **Results:** Amnion-derived cellular cytokine solution did not induce overproduction of any cytokines sampled, and there was no evidence of “cytokine storm.” It also showed no significant absorption systemically following topical delivery. No patients developed an adverse event. There were no patterns of changes in vital signs or clinical laboratory tests that were related to the treatment regimen. **Conclusion:** In this safety trial, the topical application of amnion-derived cellular cytokine solution in both intact and denuded, irradiated skin was found to be safe, and showed no evidence of systemic absorption. No cosmetic changes were identified long term. Patient blood chemistry was not adversely affected, indicating the absence of an anaphylactic response and no evidence “cytokine storm” was identified. Amnion-derived cellular cytokine solution is safe to use topically in clinical protocols.

When the skin is wounded, platelets enter the wound to control hemorrhage. The platelets release the contents of their α granules containing many growth factors that act as mitogens and chemoattractants. Platelet releasates and platelet-rich plasma have been used with some degree of success, but there is no homologous product currently available. Single growth factors made by recombinant DNA technology such as platelet-derived growth factor (PDGF), transforming growth factor beta, keratinocyte growth factor, fibroblast growth factor, insulin-like growth factor, and vascular endothelial growth factor (VEGF) have been used in clinical trials of wound healing, but only PDGF is approved for dermal wounds.^1^^,^^2^

Amnion-derived cellular cytokine solution (ACCS), made by Stemnion, Inc (Pittsburgh, Pa), is a multiple cytokine solution derived only from a unique population of naturally occurring cells isolated from the full-term placenta that is normally discarded following delivery of a baby. Each placental donor has signed an informed consent and was screened for known infectious agents. There are no living cells in ACCS, only the multiple proteins, and there has been no manipulation of the genetic cellular material. ACCS has been found to contain multiple proteins involved in wound healing including VEGF, PDGF, angiogenin, transforming growth factor β, and tissue inhibitor of metalloprotease (TIMP)-1 and TIMP-2.^1^^,^^2^

Independent collaborative studies have established proof of concept to enhance wound healing and tissue repair in both elective and traumatic wounds such as burns.^2^ ACCS has been systematically tested for safety and clinical utility. Two different preclinical studies of partial-thickness burns in guinea pigs have demonstrated accelerated epithelialization with topical ACCS. In preclinical toxicity studies, a topical ACCS dose of 0.01 mL/cm^2^ was shown to be safe.^3^^-^5

There may be applications for ACCS in many different types of wounds. Improved healing in a natural physiologic manner has been demonstrated: for example, accelerated healing of surgical incisions, more rapid healing of second- and third-degree burns with decreased scarring, and reduction of the inhibition to normal healing caused by infection.^4^^-^7 Although a need exists for effective therapies to accelerate wound healing and tissue repair, attempts to develop new wound care products have largely failed. This failure may be in part attributable to a flaw in the conception of products that did not take into account that the biology of wound healing—the body's natural restorative response to tissue injury—is the end result of a complex series of interrelated cellular processes that interact in a sequential and overlapping cascade, mediated by a range of humoral messengers (eg, cytokines and growth factors) to generate resurfacing, reconstitution, and restoration of injured tissue. Normal ideal wound healing entails multiple biologic factors; that is, nature sequences a combination of cytokines and growth factors in physiologic doses working in concert. ACCS displays many promising features of natural healing and provides a physiologic “cocktail” of the essential cytokines and growth factors necessary in wound healing. The multiple cytokines in ACCS have been found to be in physiologic concentrations, similar to levels found in normal human serum.^1^ Their effectiveness appears to be related to the fact that cytokines act in concert with each other. Traditional use of a single protein to stimulate wound healing such as PDGF, for example, must be provided in concentrations much higher that than found in nature.^8^ ACCS has been used in a phase I safety trial (IND 13,895) of partial-thickness burns and was thought to be safe when used in that trial.^9^

## MATERIALS AND METHODS

Twenty female patients with early-stage breast cancer between the ages of 18 and 80 years and who required postoperative radiotherapy were enrolled in 2 separate but related cohorts of an institutional review board–approved phase I protocol. Patients were excluded if they had abnormal hepatic or renal functions indices (minimum of 2× normal), were on hemodialysis, or were pregnant or lactating postpartum. Cohort 1 consisted of 10 patients who received topical ACCS to the breast immediately following the each of the first 10 fractions of whole breast radiotherapy. Cohort 2 consisted of 10 additional patients who fit the same criteria as the initial cohort but received topical ACCS following the development of at least grade I breast erythema as defined by the Common Terminology Criteria for Adverse Events^10^ as a consequence of the whole breast radiotherapy. Both cohorts of patients received 4.0 mL of either ACCS or 0.9% normal saline solution applied to either the lateral or medial breast (determined by double-blinded randomization) via an aerosolized spray. The surface of the breast was divided daily by drawing 2 parallel lines with a semipermanent marker vertically through the nipple-areolar complex; 1.0-cm apart. The blinded solution application was restricted to each individual side of the breast by using an adhesive surgical drape that was modified to allow only 1.0 cm of drape adhesion placed between the parallel lines. Blood was drawn from each subject immediately before and 1 hour after treatment with ACCS and again at 6 weeks. The plasma from each sample was tested by antibody array for the presence of proteins in ACCS, including platelet-derived growth factor BB (PDGF-BB), VEGF, angiogenin, TIMP-1, TIMP-2, matrix metallopeptidase (MMP-9), epidermal growth factor, and albumin. Also measured were serum levels of 9 cytokines involved in inflammation: interferon γ (IFN-γ), interleukin (IL)-1β, IL-2, IL-4, IL-6, IL-8, IL-10, IL-12p70, and tumor necrosis factor α (TNF-α). Hematologic indices were also obtained including hemoglobin (Hb), hematocrit (HCT), white blood cell (WBC) count, creatinine, bilirubin, alanine aminotransferase, aspartate aminotransferase, alkaline phosphatase, glucose, and albumin. Patients were then observed weekly with breast photography and clinical examination. An extensive quality-of-life patient questionnaire (>50 questions related to general, site-specific, and both short- and long-term features including a self-assessment of the breast cosmesis using the Harvard/National Surgical Breast and Bowel Project Cosmesis Criteria^11^) was obtained during treatment and then at 3-month intervals for a total posttreatment period of 12 months. The Harvard/National Surgical Breast and Bowel Project scale was also recorded by the physician investigators.

Before entering the study, the subjects underwent lumpectomy for early-stage breast cancer. Whole breast irradiation was instituted between 4 and 12 weeks postlumpectomy. For patients receiving chemotherapy, whole breast irradiation began 2 to 4 weeks following the final cycle of chemotherapy. No patient received neoadjuvant chemotherapy. The whole breast irradiation dose was either 50 Gy at 2.0 Gy per fraction or 50.4 Gy at 1.8 Gy per fraction. Subjects were treated using standard immobilization techniques and 3-dimensional conformal, forward planned radiotherapy. A boost to the lumpectomy cavity plus margin of 10 to 16.6 Gy in 5 to 9 fractions was used at the discretion of the radiation oncologist.

### Statistical methods

#### Safety analysis

All safety data, including vital signs, were reported and selected data were by descriptive statistics. Summaries were prepared for the number (percentage) of patients with adverse events (AEs) and serious adverse events (SAEs). Quality of life was assessed globally as well as locally by noting changes in both the medial and lateral aspects of breast including breast texture (thickening and hardness), pain, tenderness, shape, sensitivity, swelling, redness, itching, flaking skin, blistering, and fluid leak.

#### Efficacy analysis

Planned statistical hypothesis testing comparing ACCS and saline across treatment regimens was not done because the small sample size would result in limited power to detect even moderately large treatment effects. We did record what were felt to be noted differences in the separate sides of each breast when differences did occur. These will be reviewed subsequently.

## RESULTS

The primary endpoint of this safety and efficacy trial was to determine the safety profile of ACCS as measured by AEs and SAEs. The secondary endpoints were quality of life, measuring the time until the onset of erythema (inflammation), reduction in erythema in comparison of each side, reduction in moist desquamation, and time to healing comparing skin treated with ACCS versus saline placebo control.

Using this assay platform, ACCS did not activate peripheral blood mononuclear cells (PBMCs), affect their viability, or induce proliferation over a 72-hour culture period. Furthermore, the analysis of culture supernatants indicated ACCS did not induce overproduction of any cytokines associated with “cytokine storm” (IFN-γ, IL-1β, IL-2, IL-4, IL-5, IL-6, IL-8, IL-10, IL-12p70, granulocyte-macrophage colony-stimulating factor, and TNF-α) measured at the same time-points (Tables 1 and 2). In both cohorts of this clinical trial, there was no evidence of cytokine storm induction in any of the patients evaluated. Only 2 cytokines in cohort 1 (IFN-γ and IL-2) had significant elevation from pretreated plasma samples yet still did not differ substantively from levels found in pooled normal plasma samples (Tables 1 and 2). These data indicate that ACCS did not participate in the induction of cytokines associated with cytokine storm syndrome. Likewise, there were no significant differences between pre- and post-ACCS treatment PBMC expression in the activation markers CD25 and CD69. Considering T-cell proliferation is a hallmark of an immune response, these data indicate ACCS is not immunogenic. An additional relevant finding of this safety trial was that ACCS showed no significant absorption systemically following topical delivery (Table 3). Patient blood/hematology/chemistry (including total lymphocytes, monocytes, eosinophils, neutrophils, basophils, platelets, Hb, red blood cells, and HCT) were not adversely affected, indicating the absence of an anaphylactic response. One patient required coronary artery bypass 3 months following treatment that was felt to be related to long-established coronary arterial disease. There was no precipitating thrombotic event. A second patient underwent hepatic transplantation 6 weeks posttreatment for an incidentally discovered hepatocellular carcinoma, which was not known prior to treatment completion. Overall, none of the treatment-emergent AEs, AEs, or SAEs were considered to be related to treatment. No patients died or discontinued treatment due to AEs. There were no patterns of change in vital signs or clinical laboratory tests that were related to the treatment regimen.

Adverse events were nearly evenly distributed between ACCS-treated and saline-treated areas of the skin of the breast. There were no patterns of concern upon review of the AEs (Table 4). Of the AEs, 53% were mild, 38% were moderate, and 9% were severe (grade III epidermitis). There was no difference noted in time to onset of radiation-induced erythema, severity of erythema, or skin breakdown between the ACCS-treated and the saline-treated sides of the breast. There were no statistically significant changes in the serum levels of 7 proteins found to be in ACCS (Table 3). There were no significant changes in blood pressure, pulse, or temperature during the 2 weeks of drug therapy. Similarly, there were no significant changes in Hb, HCT, WBC count, serum creatinine, liver function studies (including bilirubin and hepatocellular enzymes), or glucose or albumin levels before and after the first dose of ACCS and after the 10th dose of ACCS.

## DISCUSSION

### The clinical nature of the problem

Acute radiation dermatitis is a frequent complication of whole breast radiotherapy. When the degree of dermatitis exceeds grade II as defined by the Common Terminology Criteria for Adverse Events,^10^ patients not only experience pain but also tissue compromise can lead to bleeding and infection as well as delay in treatment. To date, no single topical or systemic product has been shown to be effective in preventing this dermatitis and preparations commonly used after dermatitis begins generally result in symptomatic relief alone. Grade III and IV complications in the skin can subject patients to opportunistic dermal infections, worsening pain, and treatment delays, which sometimes can lead to lower tumor control rates.^12^^-^14

The use of ACCS has shown healing properties in some preclinical and clinical studies.^1^^,^^2^^,^^4^^-^7 As well, ACCS has been shown to be anti-inflammatory in preclinical trials at the level of potent steroids such as clobetasol (D.L.S., oral communication, 2015). However, safety in humans prior to this study has not been established; therefore, no human data exist to date to determine efficacy in the clinical situation.

There are no comprehensive guidelines on the approaches required for immunogenicity testing during product development. Likewise, there is no single source of requirements for nonclinical or clinical studies and no specific guidance for interpreting assays. Therapeutic proteins are frequently immunogenic in animals; however, immunogenicity in animal models is not always predictive of immunogenicity in humans. Animal studies may not be helpful in determining the propensity of humans for hypersensitivity responses to protein therapeutics. However, assessment of immunogenicity in animals may be useful to interpret nonclinical toxicology and pharmacology data. In addition, immunogenicity in animal models may reveal potential antibody-related toxicities that could be monitored in clinical trials. It is essential to adopt an appropriate strategy for the development of adequate screening and confirmatory assays to measure an immune response against a therapeutic protein(s). Assays should be capable of distinguishing neutralizing from non-neutralizing antibodies, be validated, and standardized.

Consequences of antibody response to a biologic treatment modality fall into 3 categories: hypersensitivity reactions, reduction in efficacy (not considered in this phase I study), and induction of autoimmune diseases. First, hypersensitivity would involve IgE antibody production following administration of a protein therapeutic. Anaphylactic findings of hypersensitivity are easily identifiable by complete blood cell count blood work in a phase I study,^15^ and this was not noted in this study. Second, cross-reactivity resulting in autoimmunity occurs when there is an antibody produced to a biologic agent that cross-reacts to a native protein (breaking tolerance), which has occurred in erythropoietin, IFN-γ, and Factor VIII trials.^16^^,^^17^ Antibody responses such as these generally occur after months of long-term treatment and usually by routes of administration involving parenteral administration. The route of administration itself plays a key role in immunogenicity of biologics, with subcutaneous treatment being associated with the highest risk or negative reaction and intravenous demonstrating one of the lowest.^18^ Topical administration is not usually even considered as any risk for antibody development to a protein therapeutic, so alternative assays of immunogenicity should be considered. Considering the topical route of administration of ACCS in this current clinical trial, immunogenicity models and experiments were developed to best determine potential immunogenic effects using in vitro and in vivo methodology, which would have to be stringently redeveloped in the case of parenteral (systemic) administration of ACCS in human subjects.

In vitro methodology using human naive PBMC effector cells were used to determine immunogenic effects of ACCS. PBMCs act as sentinels to immunogenic substances, through activation, proliferation, and cytokine production. Through these monitors, the extent of immunogenicity of a biologic protein or protein solution can be determined.^19^^,^^20^ The cascade of cytokines (mainly proinflammatory) have been associated with those documented in the syndrome known as cytokine storm.^21^ An example of this phenomenon was documented after phase I clinical trial of the monoclonal antibody TGN1412, a superagonist, which had an immediate ill-fated consequence of cytokine storm induction.^21^^-^24 Although the route of administration was systemic for this biologic, assays used to determine the detrimental cytokine storm effect were by in vitro PBMC cultures with the biologic. Using this assay platform, ACCS did not activate PBMCs, affect PBMC viability, or induce PBMC proliferation over a 72-hour culture period. Considering T-cell proliferation is a hallmark of an immune response, these data indicate that ACCS is not immunogenic. Furthermore, the analysis of culture supernatants indicate ACCS did not induce production of any of the cytokines associated with cytokine storm (IFN-γ, IL-1β, IL-2, IL-4, IL-5, IL-6, IL-8, IL-10, IL-12p70, granulocyte-macrophage colony-stimulating factor, and TNF-α) measured at the same time points. This was also seen with bound ACCS (ACCS dried to culture plates before culturing with PBMCs), which may have exposed alternate epitopes versus standard liquid drug product. These data further support the lack of immunogenicity of ACCS in vitro.

ACCS was also examined for immunogenic potential using a relevant animal model that measures sensitization to biologic compounds. This method, known as the Buehler method, is a standardized model that uses guinea pigs to measure a substance's ability to induce both an immune response in the skin and a secondary immune response upon reexposure to the test material after a period of quiescence.^25^ This type of sensitization experiment examines the potential of a protein-based therapeutic to induce a specific type of allergic response, either antibody or cell-mediated, where the immune system is activated resulting in a positive allergy effect. These effects are easily identifiable by a scoring methodology of skin irritation severity. ACCS had no effect on induction of skin irritation primarily or upon rechallenge as measured by erythema, edema, or skin irritation scoring using this methodology.^25^ This indicates ACCS did not elicit a sensitization reaction in guinea pigs. The fact that ACCS did not induce any skin irritation, even in primed test animals, indicates there was no activation of a primary or secondary immune response.

In addition, whole-blood PBMCs were analyzed for activation markers CD25 and CD69 on the same samples to examine potential posttreatment activation of immune effector cells. These markers are usually expressed on activated populations of T lymphocytes and other WBCs. An increase in expression of these markers correlates with an elevation in cell activation. Immunogenic compounds, such as proteins, can induce expression of these markers on cells over time.

Likewise, there were no significant differences between pre- and post-ACCS treatment PBMC expression in the activation markers CD25 and CD69. Together, these data are supportive in determining the safety of ACCS administered topically for treatment of inflammatory skin conditions. The fact that ACCS, when administered in this manner, is not absorbed systemically provides evidence supporting these data. Relevant findings of this safety trial including ACCS showed no significant absorption systemically following topical delivery.

## CONCLUSION

In this safety trial, the topical application of ACCS in both intact and denuded irradiated skin was found to be safe and showed no evidence of systemic absorption. There was a definite difference in the appearance of the sides of the breast in a number of women, but it did not correlate with application of ACCS following unbinding. Further investigation is warranted to evaluate the relationship of skin reaction to ACCS application due to this very stark delineation of effect. Patient blood/hematology/chemistry (including total lymphocytes, monocytes, eosinophils, neutrophils, basophils, platelets, Hb, red blood cells, and HCT) were not adversely affected, indicating the absence of an anaphylactic response. No evidence of increase of the 9 inflammatory cytokines was seen and no evidence of “cytokine storm” was identified. ACCS is safe to use topically in clinical protocols. This study has demonstrated safety, and phase II and III clinical trials may proceed from this point.

## Figures and Tables

**Table 1:** Inflammatory cytokine levels in subjects’ serum: Cohort 1

Inflammatory cytokine	Pre-ACCS treatment, mean ± SD (range)	1 h post-ACCS first treatment, mean ± SD (range)	10 d post-10th ACCS treatments, mean ± SD (range)
**IFN-γ**	9.67 ± 10.59 (2.26–38.99)	53.79 ± 133.46 (2.54–462.99)	9.63 ± 6.19 (3.74–23.02)
**IL-1β**	0.09-± 0.06 (0–0.25)	0.07 ± 0.07 (0–0.23)	0.08 ± 0.1 (0–0.3)
**IL-2**	0.17 ± 0.14 (0–0.5)	0.27 ± 0.18 (0–0.6)	0.27 ± 0.2 (0–0.75)
**IL-4**	Below detection	Below detection	Below detection
**IL-6**	2.9 ± 3.4 (0.09–10.47)	2.32 ± 2.68 (0.13–8.46)	2.01 ± 2.27 (0–8.45)
**IL-8**	65.13 ±51.72 (4.16–159.61)	14.14 ± 18.57 (1.79–68.27)	11.9 ± 6.27 (5.49–28.12)
**IL-10**	0.35 ± 0.17 (0.07–0.74)	1.4 ± 3.51 (0.06–11.86)	0.34 ± 0.19 (0–0.7)
**IL-12p70**	0.23 ± 0.23 (0–0.93)	0.24 ± 0.24 (0–0.92)	0.28 ± 0.21 (0–0.76)
**TNF-α**	7.71 ± 5.46 (0.87–17.7)	3.21 ± 1.82 (1.22–7.57)	2.87 ± 1.09 (1.23–5.27)

ACCS indicates amnion-derived cellular cytokine solution; IFN-γ, interferon γ; IL, interleukin; and TNF-α, tumor necrosis factor α.

**Table 2:** Inflammatory cytokine levels in subjects’ serum: Cohort 2

Inflammatory cytokine	Pre-ACCS treatment, mean ± SD (range)	1 h post-ACCS first treatment, mean ± SD (range)	10 d post-10th ACCS treatments, mean ± SD (range)
**IFN-γ**	6.78 ± 5.16 (2.11–17.52)	5.46 ± 2.94 (2.45–12.61)	12.61 ± 22.32 (1.23–79.03)
**IL-1β**	0.04 ± 0.02 (0–0.08)	0.04 ± 0.04 (0–0.2)	0.07 ± 0.14 (0–0.77)
**IL-2**	0.67 ± 1.31 (0–4.94)	0.54 ± 1.27 (0–4.53)	0.53 ± 1.12 (0.04–3.96)
**IL-4**	0.02 ± 0.02 (0–0.05)	0.03 ± 0.03 (0–0.18)	0.03 ± 0.03 (0–0.12)
**IL-6**	1.01 ± 0.82 (0.17–3.16)	1.37 ± 0.9 (0.19–3.07)	1.18 ± 0.67 (0–2.19)
**IL-8**	10.54 ± 7.68 (3.82–31.37)	16.01 ± 27.22 (4.43–96.67)	37.27 ± 90.5 (3.24–312.88)
**IL-10**	0.39 ± 0.53 (0.07–1.92)	0.35 ± 0.36 (0.05–1.39)	0.46 ± 0.57 (0.09–2.12)
**IL-12p70**	0.12 ± 0.08 (0–0.3)	0.15 ± 0.16 (0–0.84)	0.15 ± 0.16 (0–0.84)
**TNF-α**	2.16 ± 0.75 (0.98–4.23)	2.09 ± 0.63 (0.7–3.4)	2.49 ± 1.4 (1.04–6.55)

ACCS indicates amnion-derived cellular cytokine solution; IFN-γ, interferon γ; IL, interleukin; and TNF α, tumor necrosis factor-α.

**Table 3:** Proteins known to be in ACCS and serum: Levels in serum over time

Protein, pg/mL	Serum level baseline before first ACCS treatment, mean ± SD (range)	Serum level 1 h after first dose of ACCS, mean ± SD (range)	Serum level after 10th dose of ACCS, mean ± SD (range)
**PDGF-BB**	1,122 ± 690 (304–2,484)	924 ± 416 (223–1,867)	800 ± 422 (217–2,315)
**VEGF**	69 ± 27 (38–127)	58 ± 17 (32–88)	54 ± 18 (31–106)
**Mucin-16**	10,698 ± 7,706 (3,977–31,786)	10,964 ± 8,521 (3,645–31,344)	10,634 ± 8,013 (3,008–31,344)
**TIMP-1**	190,347 ± 53,348 (107,137–316,181)	185,138 ± 46,032 (165,477–291,013)	188,218 ± 41,599 (101,144–256,916)
**TIMP-2**	150,636 ± 60,639 (78,431–326,474)	156,218 ± 49,340 (73,637–271,201)	151,632 ± 47,003 (82,546–243,738)
**MMP-9**	164,702 ± 117,185 (33,045–465,448)	136,439 ± 96,050 (37,663–425,647)	187,683 ± 277,782 (33,284–1,283,749)
**EGF**	86 ± 41 (36–179)	75 ± 27 (22–141)	66 ± 23 (23–101)

ACCS indicates amnion-derived cellular cytokine solution; PDGF-BB, platelet-derived growth factor BB; VEGF, vascular endothelial growth factor; angiogenin, TIMP-1, TIMP-2, tissue inhibitor of metalloprotease 1 and 2; MMP-9, matrix metallopeptidase; and EGF, epidermal growth factor.

**Table 4:** Adverse events: Number observed and rate

	Treatment group				*N* = 20				
	Mild		Moderate		Severe		Total		Total
	Related*	NR	Related	NR	Related	NR	Related	NR	R + NR
Body system A (skin, breast)	0	27	0	39	0	10	0	76	76
Body system B (respiratory, mediastinum)	0	13	0	0	0	0	0	13	13
Body system C (gastrointestinal, general)	0	12	0	2	0	0	0	14	14
Body system D (other)	0	7	0	1	0	0	0	8	8

NR indicates not related; R, related.

*Related could be expanded (eg, as definite, probably, possible).

## References

[1] Steed D, Trumpower C, Duffy D (2008). Amnion-derived cellular cytokine solution. Eplasty.

[2] Uberti M, Ko F, Pierpont Y (2009). The use of amnion-derived cellular cytokine solution (ACCS) in accelerating closure of interstices in explanted meshed human skin grafts. Eplasty.

[3] Chen Z, Tortella F, Marshall D (2009). Human amnion-derived multipotent progenitor cell treatment alleviates traumatic brain injury-induced axonal degeneration. J Neurotrauma.

[4] Franz M, Payne W, Xing L (2008). The use of amnion-derived cellular cytokine solution to improve healing in acute and chronic wound models. Eplasty.

[5] Bergmann J, Hackl F, Koyama T (2009). The effect of amnion-derived cellular cytokine solution on the epithelialization of partial-thickness donor sitewounds in normal and streptozotocin-induced diabetic swine. ePlasty.

[6] Payne W, Wachtel T, Smith C, Uberti M, Ko F, Robson M (2010). Effect of amnion-derived cellular cytokine solution on healing of experimental partial-thickness burns. World J Surg.

[7] Uberti M, Lufkin A, Pierpont Y (2011). Amnion-derived cellular cytokine solution promotes macrophage activity. Ann Plast Surg.

[8] Greenhalgh DG, Sprugel K, Murray M (1990). PDGF and FGF stimulate wound healing in the genetically diabetic mouse. Am J Pathol.

[9] ClinicalTrials.gov. https://clinicaltrials.gov/ct2/show/NCT00886470.

[10] National Cancer Institute, National Institutes of Health, US Department of Health and Human Services http://evs.nci.nih.gov/ftp1/CTCAE/CTCAE_4.03_2010-06-14_QuickReference_5x7.pdf.

[11] NSABP Protocol B-39 Form COS http://rpc.mdanderson.org/rpc/credentialing/files/B39_Protocol1.pdf.

[12] Ellis F (1969). Dose, time and fractionation: a clinical hypothesis. Clin Radiol.

[13] Bese NS, Hendry J, Jeremic B (2007). Effects of prolongation of overall treatment time due to unplanned interruptions during radiotherapy of different tumor sites and practical methods for compensation. Int J Radiat Oncol Biol Phys.

[14] Bese NS, Sut PA, Ober A (2005). The effect of treatment interruptions in the postoperative irradiation of breast cancer. Oncology.

[15] Scherer K, Spoerl D, Bircher A (2010). Adverse drug reactions to biologics. J Deutchen Derm Gesellschaft.

[16] Dasgupta S, Navarrete AM, Delignat S (2007). Immune response against therapeutic factor VIII in hemophilia A patients—a survey of probable risk factors. Immunol Lett.

[17] Eckardt K, Casadevall N (2003). Pure red-cell aplasia due to anti-erythropoietin antibodies. Nephrol Dial Trans.

[18] Shankar G, Pendley C, Stein KE (2007). A risk-based bioanalytical strategy for the assessment of antibody immune responses against biological drugs. Nat Biotechnol.

[19] Worobec A, Rosenberg A (2004). Risk-based approach to immunogenicity concerns of therapeutic protein products; parts 1–3. BioPharm Int.

[20] Shankar G, Shores E, Wagner C (2006). Scientific and regulatory considerations on the immunogenicity of biologics. Trends Biotechnol.

[21] Stebbings, Findlay L, Edwards C (2007). Cytokine storm in the phase I trial of monoclonal antibody TGN1412: better understanding the causes to improve preclinical testing of immunotherapeutics. J Immunol.

[22] Attarwala H (2010). TGN1412: from discovery to disaster. J Young Pharm.

[23] Stebbings R, Poole S, Thorpe R (2009). Safety of biologics, lessons learnt from TGN1412. Cur Opin Biotechnol.

[24] Eastwood D, Findlay L, Poole S (2010). Monoclonal antibody TGN1412 trial failure explained by species differences in CD28 expression on CD4^+^ effector memory T cells. Br J Pharm.

[25] Ritz HL, Buehler EV, Drill VA, Lazar P (1980). Procedure for conducting the guinea pig assay. Current Concepts in Dermatology.

